# Crystal and mol­ecular structure of *meso*-2,6-di­bromo­hepta­nedioic acid (*meso*-2,6-di­bromo­pimelic acid)

**DOI:** 10.1107/S2056989016001754

**Published:** 2016-02-10

**Authors:** Nathaniel D. A. Dirda, Peter Y. Zavalij, Joseph P. Y. Kao

**Affiliations:** a Center for Biomedical Engineering and Technology, University of Maryland School of Medicine, Baltimore, MD 21201, USA; b Department of Chemistry and Biochemistry, University of Maryland, College Park, MD 20742, USA; c Department of Physiology, University of Maryland School of Medicine, Baltimore, MD 21201, USA

**Keywords:** crystal structure, hydrogen bonding, halogen bonding

## Abstract

The mol­ecular structure of the title compound, C_7_H_10_Br_2_O_4_, confirms the *meso* (2*R*,6*S*) configuration. In the crystal, mol­ecules are linked by pairs of O—H⋯O=C hydrogen bonds between their terminal carboxyl groups in an *R*_2_^2^(8) motif, forming extended chains that propagate parallel to the *c* axis. Adjacent chains are linked by C=O⋯Br halogen bonds.

## Abstract 2

The mol­ecular structure of the title compound, confirms the *meso*-(2*R*,6*S*) configuration. In the crystal, mol­ecules are linked by pairs of O—H⋯O=C hydrogen bonds, forming chains parallel to the *c* axis. Adjacent chains are linked by C=O⋯Br halogen bonds.

## Chemical context   

*meso*-2,6-Di­bromo­pimelic acid is a convenient starting point for preparing derivatives 2,6-disubstituted with non-halogen functional groups (for examples: Schotte, 1956*b* ▸; Lingens, 1960 ▸; Yuan & Lu, 2009 ▸). It also has utility in the synthesis of heterocycles (Schotte, 1956*b* ▸; Miyake *et al.*, 2000 ▸; Peters *et al.*, 2006 ▸; Hamon *et al.*, 2007 ▸). In an ongoing study of di­sulfides, the title compound was required as precursor to *meso*-3,7-dicarb­oxy-1,2-dithiepane. Surprisingly, other than the melting point reported by Schotte (1956*a* ▸), no further analytical data have been published on the di­bromo acid. Original stereochemical assignment was based on the lack of optical activity of the acid isolated through crystallization of the acid brucine salt (Schotte, 1956*a* ▸). The need to confirm the *meso* configuration motivated the crystal structure determination.
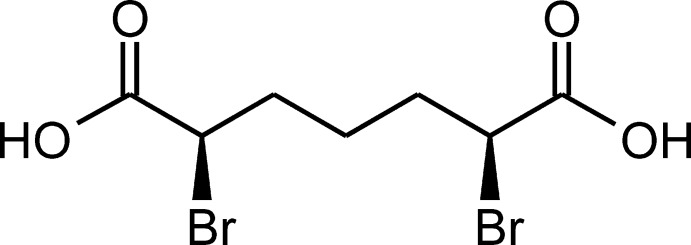


## Structural commentary   

**Figure 1:**
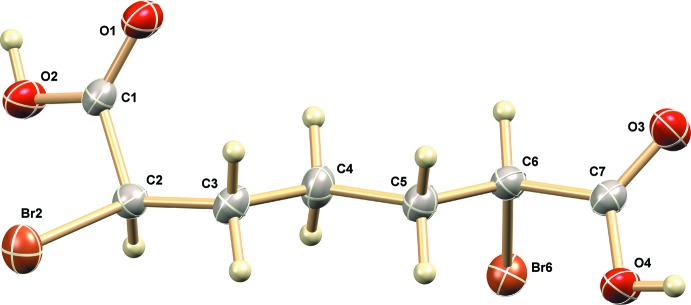
The mol­ecular structure of the title compound, with non-H atoms labeled. Displacement ellipsoids are shown at the 60% probability level.

The mol­ecular structure of the title compound is shown in Fig. 1 ▸; the (2*R*,6*S*) configuration is apparent, confirming the *meso* form of the compound. All bond lengths and angles are within normal ranges.

## Supra­molecular features   

**Figure 2:**
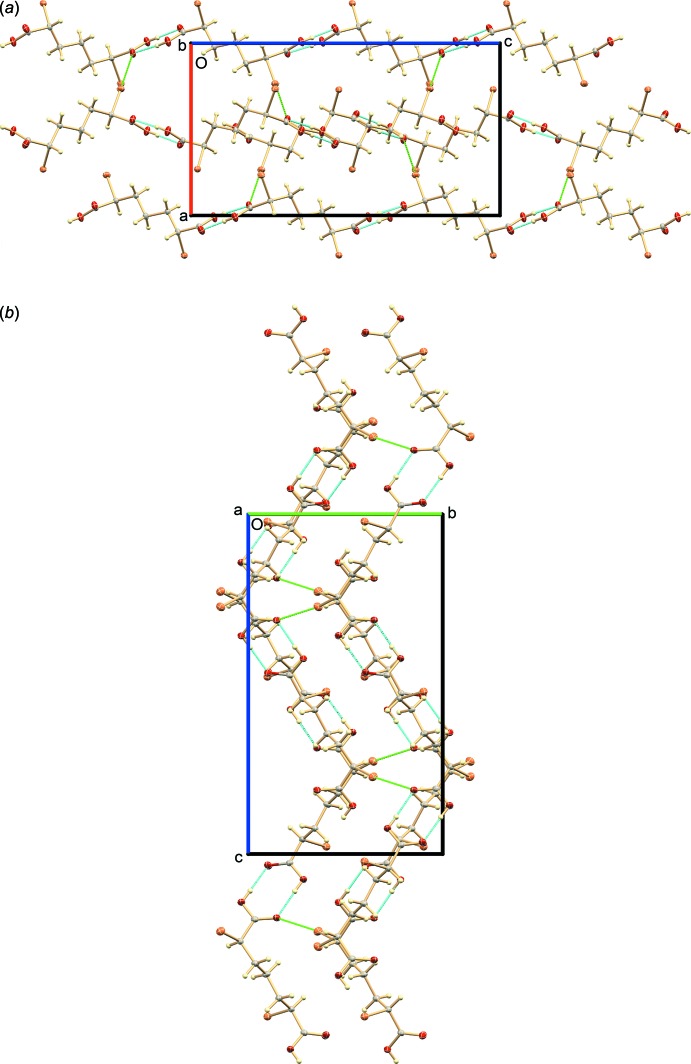
The mol­ecular packing, viewed along the *b* and *a* axes [panels (*a*) and (*b*)]. Inter­molecular hydrogen bonding (cyan) between terminal carboxyl groups results in head-to-tail linkage of the mol­ecules into chains extending along [001]. Adjacent chains are linked by halogen bonding (C=O⋯Br, green).

In the crystal, the mol­ecules are linked in head-to-tail fashion by pairs of O—H⋯O=C hydrogen bonds (Table 1 ▸) between their terminal carboxyl groups in an 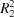
(8) motif, forming extended chains that propagate parallel to the *c* axis (Fig. 2 ▸ *a*). Adjacent chains are cross-linked by inter­actions between a carboxyl C=O group in one chain with a Br atom in an adjacent chain. These linkages meet the criteria for halogen bonds (Desiraju *et al.*, 2013 ▸): (i) the =O⋯Br—C bonds are nearly linear [the =O1⋯Br2—C2 and =O3⋯Br6—C6 angles being 168.06 (8) and 170.26 (8)°, respectively], and (ii) the O⋯Br distances [3.224 (2) and 3.058 (2) Å for O1⋯Br2^iii^ and O3⋯Br6^iv^, respectively [symmetry codes: (iii) 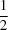 − *x*, *y* − 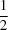, *z;* (iv) 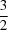 − *x*, *y* − 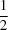, *z*] are less than the sum of the van der Waals radii of 3.35 Å (Mantina *et al.*, 2009 ▸; Alvarez, 2013 ▸). H and Br bonding are shown in Fig. 2 ▸.

## Synthesis and crystallization   

The title compound was first prepared in pure form and its stereochemistry deduced by Fehnel & Oppenlander (1953 ▸). The synthesis for the present work followed the method of Schotte (1956*a* ▸). Pimelic (hepta­nedioic) acid was converted into the diacid chloride by heating with thionyl chloride. Removal of excess SOCl_2_ under reduced pressure left the liquid diacid chloride. Over 1 h, bromine (2.3 equivalents) was added dropwise to the stirred diacid chloride maintained at 363 K. Thereafter, stirring and heating continued for an additional hour. The dibrominated acid chloride was hydrolyzed by gradual addition to vigorously stirred formic acid maintained at 353–363 K. When gas evolution ceased, the reaction mixture was refluxed for 15 min, and then allowed to cool to room temperature. Upon cooling in the refrigerator, over two days, the reaction mixture yielded two crops of solids, which were combined and extracted by shaking with ice-cold CHCl_3_. The remaining solids were recrystallized three times from formic acid to give *meso*-2,6-dibrohepta­nedioic acid (26% yield).

The ^1^H NMR spectrum, acquired in Me_2_SO-*d*_6_, is consistent with the mol­ecular structure, with the following resonances (δ referenced to Me_4_Si): 13.22, singlet, 2H; 4.43, triplet, 2H, *J* = 7 Hz; 2.01, multiplet, 2H; 1.88, multiplet, 2H; 1.54, multiplet, 1H; 1.39, multiplet, 1H. The high-resolution mass spectrum (electrospray) showed the expected manifold arising from the two stable isotopes of bromine, with the base peak at *m*/*z* = 316.884; species containing halogens other than bromine were not observed. To produce crystals suitable for diffraction, 10 mg of the title compound was dissolved in a capped glass vial in minimal formic acid with warming. Once a few seeds became visible, slow evaporation of the solvent over 14 days yielded crystals of good quality.

## Refinement details   

Crystal data, data collection and structure refinement details are summarized in Table 2 ▸. H-atom *U*_iso_ parameters were refined to confirm proper positioning of the H atoms; this was particularly important for the carboxyl H atoms. Uniquely for H3, its *U*_iso_ [0.013 (7)] is smaller than the *U*_eq_ of C3 [0.022 (4)], to which it is attached, but by less than two s.u.’s. All other H-atom *U*_iso_ values are consistent with expectation: 0.02–0.3 for CH and CH_2_, and 0.05 for CO_2_H. These values are in line with H-atom *U*_iso_ values in C_2_–C_12_ aliphatic acids without heavy-atom substitution, whose structures had been determined at the same temperature (150 K) or lower (Thalladi *et al.*, 2000 ▸; Mitchell *et al.*,2001 ▸; Peppel *et al.*, 2015*a* ▸,*b* ▸; Sonneck *et al.*, 2015*a* ▸,*b* ▸). In these structures, *U*_iso_ values average 0.033±0.006 for CH and CH_2_, and 0.068±0.033 for reciprocally hydrogen-bonded CO_2_H.

Residual electron density is somewhat high (Δρ_max_ and Δρ_min_ being 2.07 and −1.14 e Å^3^, respectively) and localizes near the heavier Br atoms, which suggests Fourier truncation as a possible cause. Other reasons could be translational pseudosymmetry (for example, see Kiessling & Zeller, 2011 ▸), or the high geometric anisotropy of the crystal (ratio of largest-to-smallest dimensions being 4), which can yield less accurate absorption correction performed through *SADABS* software. The irregular shape of the crystal precluded more accurate absorption correction through face indexing.

## Supplementary Material

Crystal structure: contains datablock(s) I. DOI: 10.1107/S2056989016001754/pk2573sup1.cif

Structure factors: contains datablock(s) I. DOI: 10.1107/S2056989016001754/pk2573Isup2.hkl

Supporting information file. DOI: 10.1107/S2056989016001754/pk2573Isup3.cml

CCDC reference: 1450356

Additional supporting information:  crystallographic information; 3D view; checkCIF report

## Figures and Tables

**Table 1:** Hydrogen-bond geometry (Å, °)

*D*—H⋯*A*	*D*—H	H⋯*A*	*D*⋯*A*	*D*—H⋯*A*
O2—H2⋯O3^i^	0.84	1.80	2.635 (3)	177
O4—H4⋯O1^ii^	0.84	1.83	2.669 (3)	176

Symmetry codes: (i) 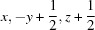; (ii) 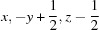.

**Table 2:** Experimental details

Crystal data	
Chemical formula	C_7_H_10_Br_2_O_4_
*M*_r_	317.97
Crystal system, space group	Orthorhombic, *P* *b* *c* *a*
Temperature (K)	150
*a*, *b*, *c* (Å)	10.4277 (7), 10.7014 (7), 18.7154 (13)
*V* (Å^3^)	2088.5 (2)
*Z*	8
Radiation type	Mo *K*α
μ (mm^−1^)	7.74
Crystal size (mm)	0.35 × 0.27 × 0.09

Data collection	
Diffractometer	Bruker SMART APEXII CCD
Absorption correction	Multi-scan (*SADABS*; Sheldrick, 2008 ▸)
*T*_min_, *T*_max_	0.231, 0.498
No. of measured, independent and observed [*I* > 2σ(*I*)] reflections	35789, 4598, 3488
*R*_int_	0.037
(sin θ/λ)_max_ (Å^−1^)	0.807

Refinement	
*R*[*F* ^2^ > 2σ(*F* ^2^)], *wR*(*F* ^2^), *S*	0.036, 0.084, 1.08
No. of reflections	4598
No. of parameters	130
H-atom treatment	Only H-atom displacement parameters refined
Δρ_max_, Δρ_min_ (e Å^−3^)	2.07, −1.15

Computer programs: *APEX2*, *SAINT* and *XSHELL* (Bruker, 2010 ▸), *SHELXS97* (Sheldrick, 2008 ▸), *SHELXL2014* (Sheldrick, 2015 ▸), *Mercury* (Macrae *et al.*, 2008 ▸), *PLATON* (Spek, 2009 ▸) and *publCIF* (Westrip, 2010 ▸).

## supplementary crystallographic information

### Crystal data

**Table:**

C_7_H_10_Br_2_O_4_	*D*_x_ = 2.023 Mg m^−^^3^
*M**_r_* = 317.97	Mo *K*α radiation, λ = 0.71073 Å
Orthorhombic, *P**b**c**a*	Cell parameters from 9811 reflections
*a* = 10.4277 (7) Å	θ = 2.2–34.7°
*b* = 10.7014 (7) Å	µ = 7.74 mm^−^^1^
*c* = 18.7154 (13) Å	*T* = 150 K
*V* = 2088.5 (2) Å^3^	Plate, colourless
*Z* = 8	0.35 × 0.27 × 0.09 mm
*F*(000) = 1232	

### Data collection

**Table:**

Bruker SMART APEXII CCD diffractometer	4598 independent reflections
Radiation source: sealed tube	3488 reflections with *I* > 2σ(*I*)
Graphite monochromator	*R*_int_ = 0.037
Detector resolution: 8.333 pixels mm^-1^	θ_max_ = 35.0°, θ_min_ = 2.2°
φ and ω scans	*h* = −16→16
Absorption correction: multi-scan (*SADABS*; Sheldrick, 2008)	*k* = −17→17
*T*_min_ = 0.231, *T*_max_ = 0.498	*l* = −30→30
35789 measured reflections	

### Refinement

**Table:**

Refinement on *F*^2^	Primary atom site location: structure-invariant direct methods
Least-squares matrix: full	Secondary atom site location: difference Fourier map
*R*[*F*^2^ > 2σ(*F*^2^)] = 0.036	Hydrogen site location: inferred from neighbouring sites
*wR*(*F*^2^) = 0.084	Only H-atom displacement parameters refined
*S* = 1.08	*w* = 1/[σ^2^(*F*_o_^2^) + (0.020*P*)^2^ + 6.*P*] where *P* = (*F*_o_^2^ + 2*F*_c_^2^)/3
4598 reflections	(Δ/σ)_max_ = 0.001
130 parameters	Δρ_max_ = 2.07 e Å^−^^3^
0 restraints	Δρ_min_ = −1.14 e Å^−^^3^

### Special details

**Table:**

Geometry. All e.s.d.'s (except the e.s.d. in the dihedral angle between two l.s. planes) are estimated using the full covariance matrix. The cell e.s.d.'s are taken into account individually in the estimation of e.s.d.'s in distances, angles and torsion angles; correlations between e.s.d.'s in cell parameters are only used when they are defined by crystal symmetry. An approximate (isotropic) treatment of cell e.s.d.'s is used for estimating e.s.d.'s involving l.s. planes.

### Fractional atomic coordinates and isotropic or equivalent isotropic displacement parameters (Å^2^)

**Table:**

	*x*	*y*	*z*	*U*_iso_*/*U*_eq_	
O1	0.4428 (2)	0.35337 (18)	0.81180 (10)	0.0271 (4)	
O2	0.5097 (2)	0.53602 (18)	0.85748 (10)	0.0289 (4)	
H2	0.5328	0.4903	0.8916	0.044 (11)*	
C1	0.4560 (2)	0.4669 (2)	0.80775 (12)	0.0200 (4)	
C2	0.4148 (2)	0.5420 (2)	0.74305 (12)	0.0190 (4)	
H2A	0.4858	0.6007	0.7303	0.024 (8)*	
Br2	0.26550 (3)	0.64147 (3)	0.77267 (2)	0.02812 (7)	
C3	0.3835 (2)	0.4638 (2)	0.67757 (12)	0.0221 (4)	
H3A	0.3411	0.5169	0.6413	0.022 (8)*	
H3B	0.3233	0.3963	0.6910	0.013 (7)*	
C4	0.5050 (3)	0.4068 (3)	0.64587 (13)	0.0241 (5)	
H4A	0.5692	0.4736	0.6380	0.030 (9)*	
H4B	0.5417	0.3462	0.6802	0.033 (9)*	
C5	0.4780 (2)	0.3408 (2)	0.57527 (13)	0.0212 (4)	
H5A	0.4112	0.2765	0.5831	0.034 (9)*	
H5B	0.4435	0.4024	0.5408	0.037 (10)*	
C6	0.5957 (2)	0.2790 (2)	0.54304 (13)	0.0204 (4)	
H6A	0.6299	0.2163	0.5778	0.029 (9)*	
Br6	0.73130 (2)	0.40137 (2)	0.52174 (2)	0.02402 (6)	
C7	0.5646 (2)	0.2132 (2)	0.47354 (12)	0.0204 (4)	
O3	0.5852 (2)	0.10197 (18)	0.46621 (11)	0.0296 (4)	
O4	0.5102 (2)	0.28358 (18)	0.42491 (10)	0.0300 (4)	
H4	0.4863	0.2389	0.3905	0.053 (12)*	

### Atomic displacement parameters (Å^2^)

**Table:**

	*U*^11^	*U*^22^	*U*^33^	*U*^12^	*U*^13^	*U*^23^
O1	0.0382 (10)	0.0244 (9)	0.0188 (8)	−0.0048 (8)	−0.0049 (7)	0.0012 (7)
O2	0.0425 (11)	0.0238 (9)	0.0205 (8)	−0.0040 (8)	−0.0098 (8)	0.0008 (7)
C1	0.0205 (10)	0.0243 (11)	0.0152 (9)	0.0001 (8)	0.0009 (7)	0.0002 (8)
C2	0.0185 (9)	0.0204 (10)	0.0181 (10)	0.0006 (8)	0.0003 (7)	0.0012 (8)
Br2	0.02097 (11)	0.03372 (14)	0.02965 (13)	0.00479 (10)	0.00001 (10)	−0.00452 (10)
C3	0.0233 (11)	0.0270 (11)	0.0159 (9)	−0.0003 (9)	−0.0012 (8)	0.0008 (8)
C4	0.0264 (11)	0.0294 (12)	0.0166 (9)	0.0032 (9)	−0.0013 (8)	−0.0036 (9)
C5	0.0226 (11)	0.0239 (11)	0.0171 (9)	0.0003 (8)	−0.0008 (8)	−0.0005 (8)
C6	0.0255 (11)	0.0190 (10)	0.0169 (9)	0.0006 (8)	−0.0024 (8)	0.0009 (8)
Br6	0.02087 (10)	0.02733 (12)	0.02386 (11)	−0.00204 (9)	−0.00007 (9)	−0.00097 (9)
C7	0.0218 (10)	0.0228 (10)	0.0165 (9)	−0.0007 (8)	−0.0008 (8)	0.0014 (8)
O3	0.0387 (11)	0.0247 (9)	0.0253 (9)	0.0061 (8)	−0.0103 (8)	−0.0034 (7)
O4	0.0493 (12)	0.0215 (8)	0.0193 (8)	0.0027 (8)	−0.0100 (8)	−0.0001 (7)

### Geometric parameters (Å, º)

**Table:**

O1—C1	1.225 (3)	C4—H4A	0.9900
O2—C1	1.314 (3)	C4—H4B	0.9900
O2—H2	0.8400	C5—C6	1.520 (3)
C1—C2	1.516 (3)	C5—H5A	0.9900
C2—C3	1.519 (3)	C5—H5B	0.9900
C2—Br2	1.966 (2)	C6—C7	1.515 (3)
C2—H2A	1.0000	C6—Br6	1.968 (2)
C3—C4	1.526 (4)	C6—H6A	1.0000
C3—H3A	0.9900	C7—O3	1.217 (3)
C3—H3B	0.9900	C7—O4	1.310 (3)
C4—C5	1.524 (3)	O4—H4	0.8400
			
C1—O2—H2	109.5	C5—C4—H4B	109.3
O1—C1—O2	124.2 (2)	C3—C4—H4B	109.3
O1—C1—C2	122.9 (2)	H4A—C4—H4B	108.0
O2—C1—C2	112.8 (2)	C6—C5—C4	113.3 (2)
C1—C2—C3	114.4 (2)	C6—C5—H5A	108.9
C1—C2—Br2	106.66 (16)	C4—C5—H5A	108.9
C3—C2—Br2	110.85 (16)	C6—C5—H5B	108.9
C1—C2—H2A	108.2	C4—C5—H5B	108.9
C3—C2—H2A	108.2	H5A—C5—H5B	107.7
Br2—C2—H2A	108.2	C7—C6—C5	111.7 (2)
C2—C3—C4	110.8 (2)	C7—C6—Br6	106.84 (16)
C2—C3—H3A	109.5	C5—C6—Br6	111.78 (16)
C4—C3—H3A	109.5	C7—C6—H6A	108.8
C2—C3—H3B	109.5	C5—C6—H6A	108.8
C4—C3—H3B	109.5	Br6—C6—H6A	108.8
H3A—C3—H3B	108.1	O3—C7—O4	124.1 (2)
C5—C4—C3	111.6 (2)	O3—C7—C6	120.9 (2)
C5—C4—H4A	109.3	O4—C7—C6	114.9 (2)
C3—C4—H4A	109.3	C7—O4—H4	109.5
			
O1—C1—C2—C3	−13.0 (3)	C3—C4—C5—C6	−178.1 (2)
O2—C1—C2—C3	165.5 (2)	C4—C5—C6—C7	179.3 (2)
O1—C1—C2—Br2	109.9 (2)	C4—C5—C6—Br6	−61.1 (2)
O2—C1—C2—Br2	−71.6 (2)	C5—C6—C7—O3	−122.1 (3)
C1—C2—C3—C4	−70.5 (3)	Br6—C6—C7—O3	115.4 (2)
Br2—C2—C3—C4	168.86 (17)	C5—C6—C7—O4	55.2 (3)
C2—C3—C4—C5	−173.3 (2)	Br6—C6—C7—O4	−67.3 (2)

### Hydrogen-bond geometry (Å, º)

**Table:**

*D*—H···*A*	*D*—H	H···*A*	*D*···*A*	*D*—H···*A*
O2—H2···O3^i^	0.84	1.80	2.635 (3)	177
O4—H4···O1^ii^	0.84	1.83	2.669 (3)	176

Symmetry codes: (i) *x*, −*y*+1/2, *z*+1/2; (ii) *x*, −*y*+1/2, *z*−1/2.

## References

[bb1] Alvarez, S. (2013). *Dalton Trans.* **42**, 8617–8636. 10.1039/c3dt50599e 23632803

[bb2] Bruker (2010). *APEX2*, *SAINT* and *XSHELL*. Bruker AXS Inc., Madison, Wisconsin, USA.

[bb3] Desiraju, G. R., Ho, P. S., Kloo, L., Legon, A. C., Marquardt, R., Metrangolo, P., Politzer, P., Resnati, G. & Rissanen, K. (2013). *Pure Appl. Chem.* **85**, 171–1713.

[bb4] Fehnel, E. A. & Oppenlander, G. C. (1953). *J. Am. Chem. Soc.* **75**, 4660–4663.

[bb5] Hamon, C., Schwarz, J., Becker, W., Kienle, S., Kuhn, K. & Schäfer, J. (2007). Int. Patent Appl. WO2007012849.

[bb6] Kiessling, A. & Zeller, M. (2011). *Acta Cryst.* E**67**, o733–o734. 10.1107/S1600536811006428 PMC3051951 21522473

[bb7] Lingens, F. (1960). *Z. Naturforsch. Teil B*, **15**, 811–811.

[bb8] Macrae, C. F., Bruno, I. J., Chisholm, J. A., Edgington, P. R., McCabe, P., Pidcock, E., Rodriguez-Monge, L., Taylor, R., van de Streek, J. & Wood, P. A. (2008). *J. Appl. Cryst.* **41**, 466–470.

[bb9] Mantina, M., Chamberlin, A. C., Valero, R., Cramer, C. J. & Truhlar, D. G. (2009). *J. Phys. Chem. A*, **113**, 5806–5812. 10.1021/jp8111556 PMC3658832 19382751

[bb10] Mitchell, C. A., Yu, L. & Ward, M. D. (2001). *J. Am. Chem. Soc.* **123**, 10830–10839. 10.1021/ja004085f 11686684

[bb11] Miyake, Y., Takada, H., Ohe, K. & Uemura, S. (2000). *J. Chem. Soc. Perkin Trans. 1*, pp. 1595–1599.

[bb12] Peppel, T., Sonneck, M., Spannenberg, A. & Wohlrab, S. (2015*a*). *Acta Cryst.* E**71**, o316. 10.1107/S2056989015007203 PMC4420089 25995924

[bb13] Peppel, T., Sonneck, M., Spannenberg, A. & Wohlrab, S. (2015*b*). *Acta Cryst.* E**71**, o323. 10.1107/S2056989015007380 PMC4420068 25995928

[bb14] Peters, D., Timmermann, D. B., Olsen, G. M., Nielsen, E. O. & Jørgensen, T. D. (2006). Int. Patent Appl. WO2006087306.

[bb15] Schotte, L. (1956*a*). *Ark. Kemi*, **9**, 407–412.

[bb16] Schotte, L. (1956*b*). *Ark. Kemi*, **9**, 413–421.

[bb17] Sheldrick, G. M. (2008). *Acta Cryst.* A**64**, 112–122. 10.1107/S0108767307043930 18156677

[bb18] Sheldrick, G. M. (2015). *Acta Cryst.* C**71**, 3–8.

[bb19] Sonneck, M., Peppel, T., Spannenberg, A. & Wohlrab, S. (2015*a*). *Acta Cryst.* E**71**, o426–o427. 10.1107/S2056989015009469 PMC4459382 26090206

[bb20] Sonneck, M., Peppel, T., Spannenberg, A. & Wohlrab, S. (2015*b*). *Acta Cryst.* E**71**, o528–o529. 10.1107/S2056989015011937 PMC4518913 26279945

[bb21] Spek, A. L. (2009). *Acta Cryst.* D**65**, 148–155. 10.1107/S090744490804362X PMC2631630 19171970

[bb22] Thalladi, V. R., Nüsse, M. & Boese, R. (2000). *J. Am. Chem. Soc.* **122**, 9227–9236.

[bb23] Westrip, S. P. (2010). *J. Appl. Cryst.* **43**, 920–925.

[bb24] Yuan, B. & Lu, S. (2009). Chin. Patent Appl. CN101497626.

